# Finite Beam Element for Curved Steel–Concrete Composite Box Beams Considering Time-Dependent Effect

**DOI:** 10.3390/ma13153253

**Published:** 2020-07-22

**Authors:** Guang-Ming Wang, Li Zhu, Xin-Lin Ji, Wen-Yu Ji

**Affiliations:** School of Civil Engineering, Beijing Jiaotong University, Beijing 100044, China; 14115279@bjtu.edu.cn (G.-M.W.); 19121058@bjtu.edu.cn (X.-L.J.); wyji@bjtu.edu.cn (W.-Y.J.)

**Keywords:** curved steel–concrete composite box beam, two-node finite beam element with 26 DOFs, long-term behavior, age-adjusted effective modulus method

## Abstract

Curved steel–concrete composite box beams are widely used in urban overpasses and ramp bridges. In contrast to straight composite beams, curved composite box beams exhibit complex mechanical behavior with bending–torsion coupling, including constrained torsion, distortion, and interfacial biaxial slip. The shear-lag effect and curvature variation in the radial direction should be taken into account when the beam is sufficiently wide. Additionally, long-term deflection has been observed in curved composite box beams due to the shrinkage and creep effects of the concrete slab. In this paper, an equilibrium equation for a theoretical model of curved composite box beams is proposed according to the virtual work principle. The finite element method is adopted to obtain the element stiffness matrix and nodal load matrix. The age-adjusted effective modulus method is introduced to address the concrete creep effects. This 26-DOF finite beam element model is able to simulate the constrained torsion, distortion, interfacial biaxial slip, shear lag, and time-dependent effects of curved composite box beams and account for curvature variation in the radial direction. An elaborate finite element model of a typical curved composite box beam is established. The correctness and applicability of the proposed finite beam element model is verified by comparing the results from the proposed beam element model to those from the elaborate finite element model. The proposed beam element model is used to analyze the long-term behavior of curved composite box beams. The analysis shows that significant changes in the displacement, stress and shear-lag coefficient occur in the curved composite beams within the first year of loading, after which the variation tendency becomes gradual. Moreover, increases in the central angle and shear connection stiffness both reduce the change rates of displacement and stress with respect to time.

## 1. Introduction

Curved steel–concrete composite box beams have gradually become one of the main design types for urban overpasses and ramp bridges due to their light weight, high torsional rigidity, and rapid construction. However, in contrast to straight beam bridges, typical bending–torsion coupling mechanical behavior can be found in curved beam bridges [1]. Hence, when the structure is subjected to a vertical load, such as its own weight, certain bending and torsional effects are generated, leading to torsional and distortional warping. For a steel–concrete composite structure, longitudinal and transverse slips exist at the interface between the steel beam and the concrete slab of the curved composite box beam. In addition, for large-curvature curved beams with a width-to-radius ratio greater than 1/10, the change in the radius of curvature along the radial direction of the beam and the shear-lag effect caused by the wide flange still need to be considered.

For the numerical analysis of curved beams, the use of a three-dimensional finite element model can accurately simulate its mechanical behavior. However, this modeling and analysis process is complex and requires high computer performance, and the calculation efficiency with a three-dimensional model is not as good as that with a one-dimensional model. The study of one-dimensional theoretical models of curved beams first began in the 1960s. Vlasov [2] established governing differential equations for curved beams based on the three major equations of mechanics, which laid the foundation for subsequent theoretical research. Later, some Japanese scholars [3,4,5] conducted related research on one-dimensional theoretical models for curved box beams and proposed relevant design proposals for the behavior of shear lag and distortion. At present, the main idea of the research on one-dimensional beam models of curved beams is to introduce functions with relevant degrees of freedom (DOFs) on the basis of Vlasov’s curved beam theoretical model to enable corresponding analyses.

The time-dependent (shrinkage and creep) effects of concrete have a significant influence on the mechanical behavior in steel–concrete composite beams. Due to the effects of concrete shrinkage and creep, the deformation of the structure under long-term loads is often much greater than that under short-term loads. The long-term deflection of composite beams has become a key issue for composite beam design and analysis. The authors of Reference [6] developed a two-node finite beam element with 26 DOFs for a curved composite box girder considering constrained torsion, distortion, shear lag, biaxial slip at the interface, and curvature variation along the radial direction. This paper introduces a constitutive relationship for concrete shrinkage and creep on the basis of the proposed beam finite element and further develops a new finite beam element that can consider the long-term behavior of curved composite box beams.

The shrinkage behavior of concrete is not related to its stress state; thus, shrinkage can be easily achieved by applying an initial strain to the model. The creep behavior of concrete is directly related to the stress state of concrete. Research on the long-term behavior of composite beams began in the 1970s. Bazant et al. [7] first simplified composite beams as a completely rigid interface and established a one-dimensional theoretical model of long-term behavior by using the three major equations of material mechanics. Afterward, Italian and Australian scholars became the primary researchers studying the long-term behavior of composite beams. Tarantino and Dezi used a stepwise calculation method to analyze the time-dependent effects of concrete, deduced a theoretical equation for simply supported composite beams, considering the time-dependent effects [8], and then derived theoretical equations for continuous composite beams, using the force method [9,10]. Then, Dezi et al. [11,12] adopted a single-step method (effective modulus method, average stress method, and age-adjusted effective modulus method) to consider the time-dependent effects of concrete and established a one-dimensional simplified theoretical model of the long-term behavior of simply supported and continuous composite beams. After entering the 21st century, based on the governing differential equations for composite beams that can consider the shear-lag behavior of concrete slabs, Dezi et al. [13] introduced a time-dependent constitutive model and established a one-dimensional theoretical model that can consider the shear-lag effect and the long-term behavior. Based on this model, the long-term behavior of composite beams was studied under prestressed loads [14]. With the increasing application of finite element technology in structural numerical analysis, a beam element model of composite beams was also proposed for analyzing their long-term behavior. Gara et al. [15] introduced a shape function to develop a beam element with 13 DOFs that can consider the interfacial slip in composite beams and the shear-lag effect of concrete slabs and analyzed the long-term behavior of the structure by using a stepwise calculation method. Ranzi and Bradford [16] proposed beam elements for the long-term behavior of composite beams, considering interfacial slip and concrete time-dependent effects according to the direct stiffness method and single-step method. Later, Ranzi, and Bradford [17] and Nguyen et al. [18] proposed long-term performance beam elements, considering interfacial slip and the time-dependent effects of concrete on the basis of the direct stiffness method and the stepwise calculation method. The abovementioned studies are all on beam element models that can analyze the long-term behavior of straight composite beams. Due to the complex mechanical behavior and time-dependent effect of curved composite box beams, it is challenging to develop a finite beam element for curved composite box beams that can account for constrained torsion, distortion, shear lag, interfacial biaxial slip, time-dependent effects, and curvature variation in the radial direction.

Based on the Vlasov’s one-dimensional theoretical model of curved beams, this paper introduces a torsional warping function, a distortional angle, an interfacial longitudinal and an interfacial transverse sliding function, and a shear-lag intensity function for concrete slabs and a shear-lag intensity function for steel beams and adopts the age-adjusted effective modulus method to analyze the effects of concrete creep. Moreover, this study establishes a finite beam element for curved composite box beams that can consider constrained torsion, distortion, shear lag, interfacial biaxial slip, time-dependent effects, and curvature variation in the radial direction. Finally, this study analyzes the long-term mechanical behavior of curved composite box girders by use of the developed finite beam element model.

## 2. Two-Node Finite Beam Element with 26 DOFs for Curved Composite Box Beams

### 2.1. Basic Hypotheses of the Model

The basic hypotheses made in the derivation process in this paper are as follows: (1) The sections of the concrete slab are rectangular, and the slab’s curvature is the same as that of the undeformed structure; (2) the vertical displacements of the concrete and the steel beam are the same; (3) the curvature radius of the composite beam does not change along the length of the beam; (4) the stiffness of the shear connections of the composite beam is uniformly distributed in the longitudinal and transverse directions; (5) the shear stress produced by the distortional effect is ignored; (6) shear deformation produced by bending and distortional warping is ignored; and (7) the shear-lag effect is taken into account under vertical bending.

### 2.2. Geometric Dimensions and Coordinate System of the Curved Composite Box Beam

**Figure 1:**
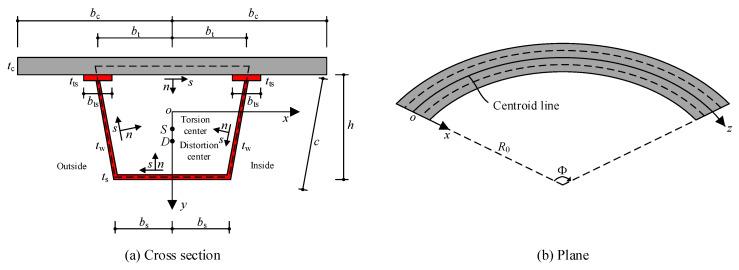
Geometric dimensions and coordinate system of the curved composite box beam: (**a**) cross section, (**b**) plane.

The geometric dimensions and coordinate system of the curved composite box beam are shown in Figure 1, wherein *oxyz* is a three-dimensional flow coordinate system that passes through the centroid line, the *ox*-axis is parallel to the concrete slab and points to the curvature center of the box beam, the *oy*-axis is straight downward, and the *oz*-axis coincides with the undeformed axis of the beam. Moreover, the tangential directions of every thin-walled bar in the sections of the box beam are set as the *s*-axis, and the normal directions of every thin-walled bar in the sections of the box beam are set as the *n*-axis.

### 2.3. Displacement Modes and Strain Components of Curved Composite Box Beams

The displacements along the *ox*, *oy*, and *oz* axes of the centroid of the curved composite box beam are denoted *u*(*z*), *v*(*z*), and *w*(*z*), respectively. The torsion angle around the torsion center and the distortion angle around the distortion center are denoted *θ*(*z*) and *θ*_d_(*z*), respectively. The transverse and longitudinal slip of the steel beam and concrete slab are denoted *a_k_*Ω*_x_*(*z*) and *a_k_*Ω*_s_*(*z*), respectively, where *k* is s or c, which represent the steel beam or concrete slab, respectively; *a*_s_ = 1 and *a*_c_ = −1; and Ω*_x_*(*z*) and Ω*_s_*(*z*) are the transverse and longitudinal slip functions, respectively. According to Ullmanski’s second torsion theory, an independent function of torsional warping displacement, *β*(*z*), is introduced. Additionally, *f_k_* (*k* = s,c) is the shear-lag warping strength functions that represent the steel beam or concrete slab. Based on the coordinate system shown in Figure 1, according to the curved shell theory, combining the introduced transverse and longitudinal slip functions and the shear-lag warping strength functions of the steel beam and concrete slab, the longitudinal displacement *W_zk_*(*z*,*s*), tangential displacement *W_sk_*(*z*,*s*), and normal displacement *W_nk_*(*z*,*s*) at any point of the composite box beam can be obtained as follows:

(1) $$\begin{array}{l} W_{zk}(z,s)=w-(u^{\prime }+wk_{0}+a_{k}\Omega_{z}k_{0})x-v^{\prime }y+a_{k}\Omega_{z}\\ -\omega (x,y)(\beta +v^{\prime }k_{0})-\omega_{d}(x,y)\theta_{d}{}^{\prime }+\psi_{k}(x)f_{k} \end{array}$$

(2) $$W_{sk}(z,s)=-(u+a_{k}\Omega_{x})\sin\alpha -v\cos\alpha +\theta \rho_{s}+\theta_{d}D_{s}$$

(3) $$W_{nk}(z,s)=(u+a_{k}\Omega_{x})\cos\alpha -v\sin\alpha -\theta \rho_{n}-\theta_{d}D_{n}$$

where *k*_0_ = 1/*R*_0_ is the initial curvature of the centroid line of the box beam sections; *R*_0_ is the curvature radius of the centroid line of the box beam sections; *α* is the angle from the *x*-axis to the *n*-axis, which is anticlockwise positive; *ω*(*x*,*y*) and *ω*_d_(*x*,*y*) are the torsional warping and the distortional warping principal polar coordinates, respectively; and *ψ_k_*(*x*) is the shape function for the shear-lag warping of the concrete slab or steel beam, the expressions of which are as shown in Appendix A.

Transformed sections should be adopted for calculating geometric characteristics. For the calculation of the axial and bending characteristics of the composite beam, the thickness of the concrete slab is unchanged, whereas the width decreases. For the calculation of torsional and distortional effects of the composite beam, the width of the concrete slab remains unchanged, whereas the thickness decreases.

In addition, according to the literature [19,20], in Equations (1)–(3), $\rho_{s}=\left(y-y_{s}\right)\sin\alpha -\left(x-x_{s}\right)\cos\alpha$ is the tangential displacement distribution of the cross-sections of the composite beam under a unit torsion angle; $\rho_{n}=\left(y-y_{s}\right)\cos\alpha +\left(x-x_{s}\right)\sin\alpha$ is the normal displacement distribution of the cross-sections of the composite beam under a unit torsion angle; $D_{s}=\left[\left(y-y_{d}\right)\sin\alpha -\left(x-x_{d}\right)\cos\alpha \right]\Psi_{d}$ is the tangential displacement distribution of the composite beam under a unit distortion angle; $D_{n}=\left[\left(y-y_{d}\right)\cos\alpha +\left(x-x_{d}\right)\sin\alpha \right]\Psi_{d}$ is the normal displacement distribution of the composite beam under a unit distortion angle; (*x*_s_, *y*_s_) and (*x*_d_, *y*_d_) are the coordinates of the torsion center, *S*, and the distortion center, *D*, of the transformed sections of the composite beam, respectively; $\Psi_{d}=-1/\left(1+\nu \right)$ is the distortion parameter of the top and bottom plates, and $\Psi_{d}=\nu /\left(1+\nu \right)$ is the distortion parameter of the web, in which $\nu =\left(2h_{1}b_{t}+2h_{3}b_{s}\right)/\left(h_{2}c+h_{4}c\right)$ is a constant related only to the section shape [20]; *h*_1_ and *h*_3_ are the vertical distances from the distortion center to the top and bottom plates, respectively; and *h*_2_ and *h*_4_ are the vertical distances from the distortion center to the left and right webs, respectively.

Note that *a*_s_ is 1 for the steel beam and −1 for the concrete slab. According to Equations (1) and (2), the relative slips on the interface between the steel beam and concrete slab are expressed as follows:

(4) $$u_{\mathrm{sp}}=2\Omega_{x}$$

(5) $$w_{\mathrm{sp}}=2\Omega_{z}$$

where *u*_sp_ and *w*_sp_ denote the transverse and longitudinal interfacial slip, respectively.

Based on the abovementioned displacement mode, according to the curved shell theory and taking the curvature variation in the radial direction of the beam into consideration, the normal strain component at any point, *P*, in the three-dimensional flow coordinate system is expressed as follows:

(6) $$\begin{array}{cc} \varepsilon_{pk} & =\frac{\partial W_{zk}}{\rho (x)\partial \phi }+\frac{W_{s}\sin\alpha }{\rho (x)}-\frac{W_{n}\cos\alpha }{\rho (x)}\\ & =\frac{R_{0}}{\rho (x)}\left(\frac{\partial W_{zk}}{R_{0}\partial \phi }+\frac{W_{s}\sin\alpha }{R_{0}}-\frac{W_{n}\cos\alpha }{R_{0}}\right)\\ & =\frac{R_{0}}{\rho (x)}[w^{\prime }-xu^{\prime \prime }-yv^{\prime \prime }+a_{k}(1-k_{0}x)\Omega_{z}{}^{\prime }-k_{0}u-k_{0}xw^{\prime }-a_{k}k_{0}\Omega_{x}+\\ & \;k_{0}(y-y_{s})\theta -\omega (\beta^{\prime }+k_{0}v^{\prime \prime })+k_{0}(y-y_{d})\Psi_{d}\theta_{d}-\omega_{d}\theta_{d}{}^{\prime \prime }+\psi_{k}f_{k}{}^{\prime }] \end{array}$$

According to Hypothesis (5), based on the curved shell theory, the shear strain component at any point, *P*, of the cross-section is expressed as follows:

(7) $$\gamma_{pk}=\frac{R_{0}}{\rho (x)}\left[(\theta^{\prime }+v^{\prime }k_{0})r^{*}-(\beta +v^{\prime }k_{0})\frac{\partial \omega }{\partial s}+\psi_{k,x}f_{k}\right]$$

where r* denotes the vertical distance between the torsion center and the tangent line of point, *P*.

(8) $$\frac{\partial \omega }{\partial s}=r^{*}-\frac{\Omega }{\tilde{t}\oint \left(ds/\tilde{t}\right)}$$

where $\Omega =\oint \tilde{t}ds$ is twice the surrounding area, and $\tilde{t}$ is the thickness of each thin-walled plate of the transformed section.

### 2.4. Equilibrium Equation for Curved Composite Box Beams

The equilibrium equation for a curved steel–concrete composite beam can be derived according to the virtual work principle as follows:

(9) $$\begin{array}{cc} \delta \Pi = & \int_{\Phi }\underset{A_{s}}{∯}\delta \varepsilon_{s}^{T}\sigma_{s}da\rho (x)d\phi +\int_{\Phi }\underset{A_{c}}{∯}\delta \varepsilon_{c}^{T}\sigma_{c}da\rho (x)d\phi +\int_{\Phi }\int_{2b_{\mathrm{ts}}}\delta d_{\mathrm{slip}}^{T}q_{\mathrm{sh}}dx\rho (x)d\phi +\\ & \int_{\Phi }\delta \theta_{d}K_{R}\theta_{d}\rho (x)d\phi -\sum \delta W^{T}Q-\int_{\Phi }\delta W^{T}q\rho (x)d\phi =0\\ & \;\forall \delta \varepsilon_{s},\delta \varepsilon_{c},\delta d_{\mathrm{slip}},\delta \theta_{d},\delta W \end{array}$$

where $\int_{\Phi }\underset{A_{s}}{∯}\delta \varepsilon_{s}^{T}\sigma_{s}da\rho (x)d\phi$ and $\int_{\Phi }\underset{A_{c}}{∯}\delta \varepsilon_{c}^{T}\sigma_{c}da\rho (x)d\phi$ are the internal virtual work produced by the deformation of the steel beam and concrete slab, respectively; As and Ac denote the cross-sectional areas of the steel beam and concrete slab, respectively; and Φ is the central angle of the curved composite box beam. In terms of Equations (6) and (7), the strain variables of the steel beam and concrete slab can be written as follows:

(10) $$\varepsilon_{k}=SB_{k}d$$

where the dimensions of the matrix ***B_k_*** are 11 × 23, and its exact value is given in Appendix B. The matrix ***S*** and displacement vector ***d*** are expressed as follows:

(11) $$S=\left[\begin{array}{ccccccccccc} \frac{R_{0}}{\rho (x)} & \frac{R_{0}}{\rho (x)}x & \frac{R_{0}}{\rho (x)}y & \frac{R_{0}}{\rho (x)}\omega & \frac{R_{0}}{\rho (x)}\omega_{d} & \frac{R_{0}}{\rho (x)}\psi_{c} & \frac{R_{0}}{\rho (x)}\psi_{s} & 0 & 0 & 0 & 0\\ 0 & 0 & 0 & 0 & 0 & 0 & 0 & \frac{R_{0}}{\rho (x)}r^{*} & \frac{\partial \omega }{\partial s} & \psi_{c,x} & \psi_{s,x} \end{array}\right]$$

(12) $$d={\left\{\begin{array}{cccc} \left[u\right] & \left[v\right] & \left[w\right] & \begin{array}{cccc} \left[\theta \right] & \left[\beta \right] & \left[\theta_{d}\right] & \begin{array}{cc} \left[\Omega \right] & \left[f\right] \end{array} \end{array} \end{array}\right\}}^{T}$$

where

(13) $$[u]=\left(\begin{array}{ccc} u & u^{\prime } & u^{\prime \prime } \end{array}\right)$$

(14) $$[v]=\left(\begin{array}{ccc} v & v^{\prime } & v^{\prime \prime } \end{array}\right)$$

(15) $$[w]=\left(\begin{array}{cc} w & w^{\prime } \end{array}\right)$$

(16) $$[\theta ]=\left(\begin{array}{cc} \theta & \theta^{\prime } \end{array}\right)$$

(17) $$[\beta ]=\left(\begin{array}{cc} \beta & \beta^{\prime } \end{array}\right)$$

(18) $$[\theta_{d}]=\left(\begin{array}{ccc} \theta_{d} & \theta_{d}{}^{\prime } & \theta_{d}{}^{\prime \prime } \end{array}\right)$$

(19) $$[\Omega ]=\left(\begin{array}{cccc} 2\Omega_{x} & 2\Omega_{x}{}^{\prime } & 2\Omega_{s} & 2\Omega_{s}{}^{\prime } \end{array}\right)$$

(20) $$[f]=\left(\begin{array}{cccc} f_{c} & f_{c}{}^{\prime } & f_{s} & {f^{\prime }}_{s} \end{array}\right)$$

When the bridge is in use, the steel beam is in the elastic phase, and the stress–strain relationship of the steel beam is expressed as follows:

(21) $$\sigma_{s}=\left\{\begin{array}{c} \sigma_{s}\\ \tau_{s} \end{array}\right\}=E_{s}\left[\begin{array}{cc} 1 & 0\\ 0 & 1/2\left(1+\mu_{s}\right) \end{array}\right]\left\{\begin{array}{c} \varepsilon_{s}\\ \gamma_{s} \end{array}\right\}$$

where *σ*_s_ and *τ*_s_ are the normal stress and shear stress of the steel beam, respectively; *E*_s_ is the elastic modulus of steel; and *μ*_s_ is the Poisson’s ratio of steel.

To take the effects of concrete shrinkage and creep into consideration, the stress–strain relationship of the concrete slab can be expressed as follows:

(22) $$\left\{\begin{array}{c} \varepsilon_{c}\left(t\right)-\varepsilon_{c}^{\mathrm{sh}}\left(t\right)\\ \gamma_{c}\left(t\right) \end{array}\right\}=J\left(t,t_{0}\right)\left[\begin{array}{cc} 1 & 0\\ 0 & 2\left(1+\mu_{c}\right) \end{array}\right]\left\{\begin{array}{c} \sigma_{c}\left(t_{0}\right)\\ \tau_{c}\left(t_{0}\right) \end{array}\right\}+\int_{t_{0}}^{t}J\left(t,\tau \right)\left[\begin{array}{cc} 1 & 0\\ 0 & 2\left(1+\mu_{c}\right) \end{array}\right]\left\{\begin{array}{c} d\sigma_{c}\left(\tau \right)\\ d\tau_{c}\left(\tau \right) \end{array}\right\}$$

To solve the integral problem in Equation (22), Bazant [21] proposed the age-adjusted effective modulus method to calculate the stress–strain relationship of concrete, which is expressed as follows:

(23) $$\sigma_{c}\left(t\right)=\left\{\begin{array}{c} \sigma_{c}\left(t\right)\\ \tau_{c}\left(t\right) \end{array}\right\}=E_{\mathrm{ce}}\left(t,t_{0}\right)\left[\begin{array}{cc} 1 & 0\\ 0 & 1/\left(2+2\mu_{c}\right) \end{array}\right]\left\{\begin{array}{c} \varepsilon_{c}\left(t\right)-\varepsilon_{c}^{\mathrm{sh}}\left(t\right)\\ \gamma_{c}\left(t\right) \end{array}\right\}+\xi \left(t,t_{0}\right)\left\{\begin{array}{c} \sigma_{c}\left(t_{0}\right)\\ \tau_{c}\left(t_{0}\right) \end{array}\right\}$$

where *t*_0_ is the initial loading age; $\varepsilon_{c}^{\mathrm{sh}}$ is the concrete shrinkage strain; *μ*_c_ is the Poisson’s ratio of concrete; *J*(*t*,*t*_0_) is the concrete creep function at time *t*; *σ*_c_ and *τ*_c_ are the normal stress and shear stress of the concrete slab, respectively; *E*_ce_(*t*,*t*_0_) is the concrete age-adjusted effective elastic modulus; and *ξ*(*t*,*t*_0_) is the influence factor of the creep effects. *E*_ce_(*t*,*t*_0_) and *ξ*(*t*,*t*_0_) can be calculated by Equations (24) and (25), respectively.

(24) $$E_{\mathrm{ce}}\left(t,t_{0}\right)=\frac{E_{c}\left(t_{0}\right)}{1+\chi \left(t,t_{0}\right)\varphi \left(t,t_{0}\right)}$$

(25) $$\xi \left(t,t_{0}\right)=\frac{\varphi \left(t,t_{0}\right)\left(\chi \left(t,t_{0}\right)-1\right)}{1+\chi \left(t,t_{0}\right)\varphi \left(t,t_{0}\right)}$$

where *E*_c_(*t*_0_) is the concrete elastic modulus at time *t*_0_, *φ*(*t*,*t*_0_) is the concrete creep coefficient, and *χ*(*t*,*t*_0_) is the concrete aging coefficient, which can be derived from Equation (26).

(26) $$\chi \left(t,t_{0}\right)=\frac{E_{c}\left(t_{0}\right)}{E_{c}\left(t_{0}\right)-R\left(t,t_{0}\right)}-\frac{1}{\varphi \left(t,t_{0}\right)}$$

where *R*(*t*,*t*_0_) is the concrete relaxation function, which can be calculated according to the literature [21].

Note that $\int_{\Phi }\int_{2b_{\mathrm{ts}}}\delta d_{\mathrm{slip}}^{T}q_{\mathrm{sh}}dx\rho (x)d\phi$ in Equation (9) is the internal virtual work produced by interfacial slip between the steel beam and concrete slab. According to Equations (4) and (5), the interfacial slip between the steel beam and concrete slab can be written as follows:

(27) $$d_{\mathrm{slip}}={\left\{\begin{array}{cc} u_{\mathrm{sp}} & w_{\mathrm{sp}} \end{array}\right\}}^{T}=S_{\Omega }B_{\Omega }d$$

where the dimensions of matrix ***B*_Ω_** are 2 × 23, as shown in Appendix B, and ***S*_Ω_** is an element matrix with dimensions of 2 × 2.

During use, the shear stress flow of a unit area at the interface between the steel beam and concrete slab can be expressed as follows:

(28) $$q_{\mathrm{sh}}={\left\{\begin{array}{cc} q_{\mathrm{us}} & q_{\mathrm{ws}} \end{array}\right\}}^{T}=\left[\begin{array}{cc} \rho_{u} & 0\\ 0 & \rho_{w} \end{array}\right]\left\{\begin{array}{c} u_{\mathrm{sp}}\\ w_{\mathrm{sp}} \end{array}\right\}=\rho_{\mathrm{slip}}d_{\mathrm{slip}}$$

where *q*_us_ and *q*_ws_ are transverse and longitudinal shear stress flow of a unit area in the composite beam interface, respectively; ***ρ*_slip_** is the shear connection stiffness matrix of a unit area in the composite beam interface; and *ρ*_u_ and *ρ*_w_ are the transverse and longitudinal shear connection stiffness of a unit area in the composite beam, respectively.

Note that $\int_{\Phi }\delta \theta_{d}K_{R}\theta_{d}\rho (x)d\phi$ in Equation (9) is the internal virtual work produced when the frame resists distortion. *K*_R_ is the frame distortion-resisting stiffness, which can be calculated according to the literature [22].

Note that $\sum \delta W^{T}Q$ and $\int_{\Phi }\delta W^{T}q\rho (x)d\phi$ in Equation (9) are the external virtual work produced by external loads, wherein $Q={\left(\begin{array}{ccc} Q_{n} & Q_{s} & Q_{z} \end{array}\right)}^{T}$ and $q={\left(\begin{array}{ccc} q_{n} & q_{s} & q_{z} \end{array}\right)}^{T}$ are the concentrated load vector and the uniformly distributed load vector, respectively; *Q_n_*, *Q_s_*, and *Q_z_* are concentrated forces in the directions of the *n*-axis, *s*-axis, and *z*-axis, respectively; *q_n_*, *q_s_*, and *q_z_* are uniformly distributed forces in the directions of the *n*-axis, *s*-axis, and *z*-axis, respectively; and ***W*** is the displacement vector under external loads. The equation for this displacement vector is given in Equation (29), which is based on Equations (1)–(3).

(29) $$W={\left\{\begin{array}{ccc} W_{nk} & W_{sk} & W_{zk} \end{array}\right\}}^{T}=H_{1}A+H_{2}A^{\prime }$$

where

(30) $${\left[H_{1}\right]}_{3\times 10}={\left\{\begin{array}{ccc} {\left[B_{1}\right]}^{T} & {\left[B_{2}\right]}^{T} & {\left[B_{3}\right]}^{T} \end{array}\right\}}^{T}$$

(31) $${\left[H_{2}\right]}_{3\times 10}={\left\{\begin{array}{ccc} {\left[0\right]}_{1\times 10}{}^{T} & {\left[0\right]}_{1\times 10}{}^{T} & {\left[B_{4}\right]}^{T} \end{array}\right\}}^{T}$$

(32) $$B_{1}=\left(\begin{array}{cccccccccc} \cos\alpha & -\sin\alpha & 0 & -\rho_{n} & 0 & -D_{n} & \frac{a_{k}\cos\alpha }{2} & 0 & 0 & 0 \end{array}\right)$$

(33) $$B_{2}=\left(\begin{array}{cccccccccc} -\sin\alpha & -\cos\alpha & 0 & \rho_{s} & 0 & D_{s} & -\frac{a_{k}\sin\alpha }{2} & 0 & 0 & 0 \end{array}\right)$$

(34) $$B_{3}=\left(\begin{array}{cccccccccc} 0 & 0 & 1-xk_{0} & 0 & -\omega & 0 & 0 & \frac{a_{k}\left(1-xk_{0}\right)}{2} & \psi_{c} & \psi_{s} \end{array}\right)$$

(35) $$B_{4}=\left(\begin{array}{cccccccccc} -x & -(y+\omega k_{0}) & 0 & 0 & 0 & -\omega_{d} & 0 & 0 & 0 & 0 \end{array}\right)$$

(36) $$A={\left(\begin{array}{cccc} \begin{array}{cccc} u & v & w & \theta \end{array} & \beta & \theta_{d} & \begin{array}{cc} 2\Omega_{x} & 2\Omega_{z}f_{c}f_{s} \end{array} \end{array}\right)}^{T}$$

### 2.5. Finite Beam Element for Curved Composite Box Beams

According to the methodology of finite element discretization, Equation (9) can be solved by adopting a two-node finite beam element, wherein thirteen DOFs exist at each of the nodes. The nodal displacement vector of the two-node finite beam element with 26 DOFs is expressed as follows:

(37) $$d^{e}={\left\{\begin{array}{cc} d_{1}^{e} & d_{2}^{e} \end{array}\right\}}^{T}$$

(38) $$d_{i}^{e}=\left(\begin{array}{cccc} \begin{array}{cccc} \begin{array}{cccc} \begin{array}{cc} u_{i} & u_{i}{}^{\prime } \end{array} & v_{i} & v_{i}{}^{\prime } & w_{i} \end{array} & \theta_{i} & \beta_{i} & \theta_{di} \end{array} & \theta_{di}{}^{\prime } & 2\Omega_{xi} & \begin{array}{ccc} 2\Omega_{zi} & f_{ci} & f_{si} \end{array} \end{array}\right)\;\mathrm{for}\;i=1,2$$

The shape function matrix [***N***]_23 × 26_ is introduced into Equation (12), and the shape function matrix [***N*_F_**]_10 × 26_ is introduced into Equation (36); both of the shape function matrices are shown in Appendix C. Substituting Equations (10)–(36) and the shape function matrices ***N*** and ***N*_F_** into Equation (9), the finite beam element equation for the curved composite box beam can be derived as follows:

(39) $$Kd^{e}=F$$

[***K***]_26 × 26_ in Equation (39) is the element stiffness matrix, which can be calculated as follows:

(40) $$K=\int_{l_{e}}N^{T}\left[\iint_{A_{s}}B_{s}^{T}S^{T}E_{s}SB_{s}da+\iint_{A_{c}}B_{c}^{T}S^{T}E_{c}SB_{c}da+\int_{2b_{\mathrm{ts}}}B_{\Omega }^{T}S_{\Omega }^{T}\rho_{\mathrm{slip}}S_{\Omega }B_{\Omega }dx+T_{R}\right]Ndz$$

where [***T*_R_**]_23 × 23_ is the distortion-resisting stiffness matrix of the frame, in which *T*_R_(13,13) = *K*_R_ and the other elements are 0, and *l*_e_ is the length of the finite beam elements.

[***F***]_26__× 1_ in Equation (39) is the equivalent load vector of the node, which can be calculated as follows:

(41) $$F=F_{q}+F_{\xi }+F_{\mathrm{sh}}$$

where ***F*_q_** is the equivalent load vector of the node caused by external loads, which can be calculated by Equation (42); ***F*_ξ_** is the equivalent load vector of the node caused by the concrete creep effect, which can be calculated by Equation (43); and ***F*_sh_** is the equivalent load vector of the node caused by the concrete shrinkage effect, which can be calculated by Equation (44).

(42) $$F_{q}=\int_{l_{e}}\left(N_{F}^{T}H_{1}^{T}+{N^{\prime }}_{F}^{T}H_{2}^{T}\right)qdz+\left(N_{F}^{T}H_{1}^{T}+{N^{\prime }}_{F}^{T}H_{2}^{T}\right)Q$$

(43) $$F_{\xi }=-\int_{l_{e}}N^{T}B_{c}{}^{T}\left(\underset{A_{c}}{∯}S^{T}\xi \left(t,t_{0}\right)\sigma_{c}\left(t_{0}\right)da\right)dz$$

(44) $$F_{\mathrm{sh}}=\int_{l_{e}}N^{T}B_{c}{}^{T}\left(\underset{A_{c}}{∯}S^{T}E_{\mathrm{ce}}\left(t,t_{0}\right)\varepsilon_{c}^{\mathrm{sh}}\left(t\right)da\right)dz$$

where

(45) $$\sigma_{c}\left(t_{0}\right)={\left\{\begin{array}{cc} \sigma_{c}(t_{0}) & \tau_{c}(t_{0}) \end{array}\right\}}^{T}$$

(46) $$\varepsilon_{c}^{\mathrm{sh}}\left(t\right)={\left\{\begin{array}{cc} \varepsilon_{c}^{\mathrm{sh}}\left(t\right) & 0 \end{array}\right\}}^{T}$$

Notably, the calculation methods for the characteristic geometrical parameters of the section under torsion and distortion in the finite beam element model can be found in the literature [6,23].

## 3. Numerical Validation of the Beam Element Model

**Figure 2:**
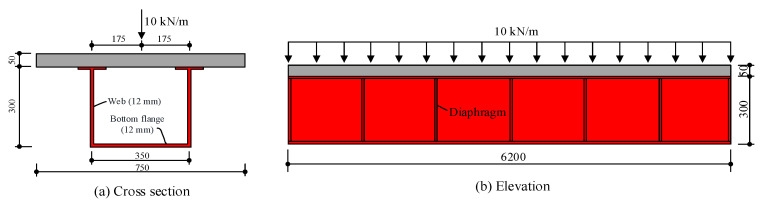
Geometric dimensions and load layout of the curved composite box beam used for validation (unit: mm): (**a**) cross section, (**b**) elevation.

The proposed finite beam element model is applied to calculate the long-term behavior of a typical curved composite box beam. The cross-section and load layout of the curved composite box beam are shown in Figure 2: The arc length of the longitudinal section centroid line is 6200 mm, the central angle is 45°, and seven transverse diaphragms are uniformly distributed along the longitudinal direction; the slab is composed of C30 concrete and is 750 mm wide and 50 mm thick; the steel beam has a total height of 300 mm; the web has a thickness of 12 mm; the bottom flange is 350 mm wide and 12 mm thick; the interfacial shear connection stiffness along the transverse and longitudinal directions, *ρ*_u_ and *ρ*_w_, are both equal to 100 N/mm^3^; and a vertical load of 10 kN/mm acts at the section centroid line.

The boundary conditions of the beam element model are as follows: At the mid-span, the longitudinal displacement and the longitudinal slip between the concrete slab and steel beam are all restrained to ensure symmetrical structural boundary constraints; at both ends of the beam, the transverse displacement, transverse slip, deflection, and torsion angle are restrained; and at the position of every diaphragm, the distortion angle is restrained.

**Figure 3:**
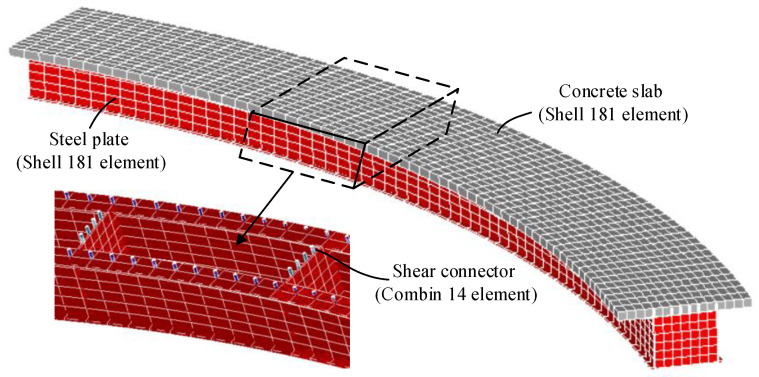
Elaborate finite element model of a curved composite box beam established in ANSYS.

The elaborate finite element model of a curved steel–concrete composite box beam is established in ANSYS. The concrete slabs, steel beams, and transverse diaphragms are modeled with Shell181 elements, whereas the shear connectors are modeled with Combin14 elements. The finite element model is shown in Figure 3.

**Figure 4:**
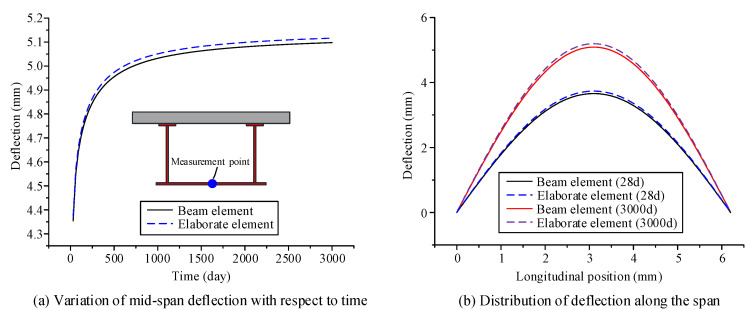
Deflection variation in curved composite box girder, with respect to time: (**a**) variation of mid-span deflection with respect to time, (**b**) distribution of deflection along the span.

**Figure 5:**
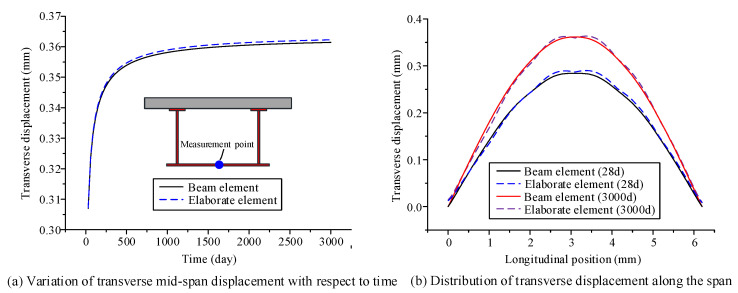
Transverse displacement variation in the curved composite box girder, with respect to time: (**a**) variation of transverse mid-span displacement with respect to time, (**b**) distribution of transverse displacement along the span.

**Figure 6:**
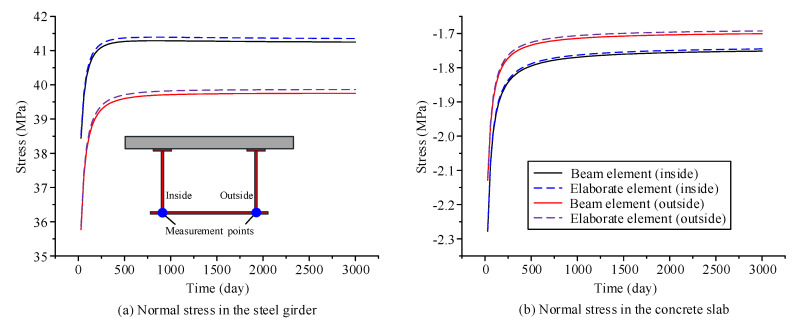
Stress variation in the steel girder and concrete slab of the curved composite box beam, with respect to time: (**a**) normal stress in the steel girder, (**b**) normal stress in the concrete slab.

The parameters of the material and environment of the proposed beam element model and the elaborate finite element model established in ANSYS are as follows: The elastic modulus of steel *E*_s_ and Poisson’s ratio of steel *μ*_s_ are 2.06 × 10^5^ MPa and 0.3, respectively; the calculation of the creep behavior of concrete in the two models use the age-adjusted effective modulus method; and the Poisson’s ratio of concrete *μ*_c_ is 0.2. The elastic modulus formula and the shrinkage creep model are obtained within CEB-FIP90 [24]; the drying age of concrete is three days, the relative humidity in the environment is 75%, and the initial loading age *t*_0_ is 28 days. Using the finite beam element model and the elaborate finite element model, time-dependent changes in the deflection, transverse displacement, and stress of the curved composite box beam were calculated from the loading age to 3000 days, as shown in Figure 4, Figure 5 and Figure 6. From these three figures, the accuracy and applicability of the proposed two-node finite element with 26 DOFs are verified due to the good correlation between the results of the elaborate finite element model and those of the finite beam element model. Notably, the proposed beam element model and the elaborate FE model took 7 and 53 s to obtain the long-term behavior of the curved composite beam, respectively. It can be known that the calculation efficiency of the proposed beam element model is much higher than that of the elaborate FE model.

According to the three figures, the deflection and transverse displacement of the curved composite box beam increase over time due to concrete shrinkage and creep, and the compressive stress in the concrete decreases over time, whereas the tensile stress in the steel beam increases over time. Within 250 days after loading, the displacement and the stress of the curved composite box beam change significantly with respect to time, and then the growth rate slows and becomes gradual after the first year. The results show that the influence of concrete shrinkage and creep is significant in the early loading stage, whereas this influence is very small after the first year.

## 4. Parametric Analysis

### 4.1. Influence of the Time-Dependent Effects on Shear Lag

**Figure 7:**
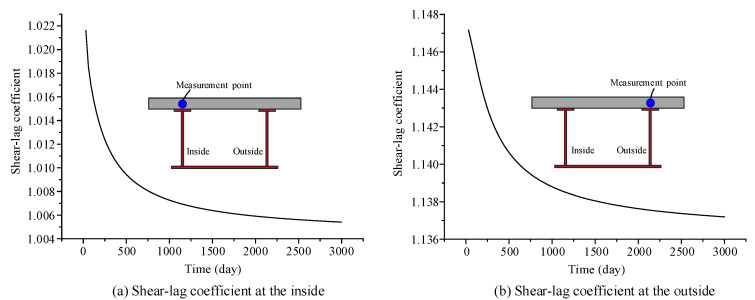
Change in the shear-lag warping strength function of the concrete slab, with respect to time: (**a**) shear-lag coefficient at the inside; (**b**) shear-lag coefficient at the outside.

**Figure 8:**
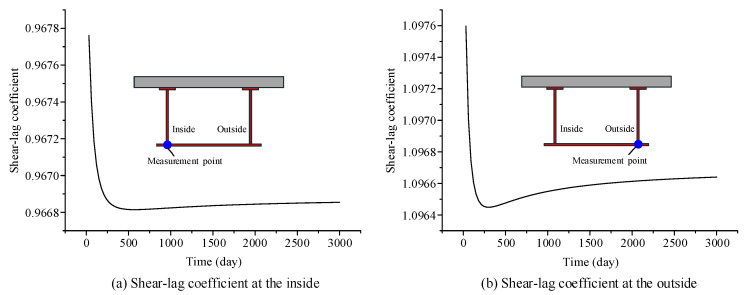
Change in the shear-lag warping strength function of the steel bottom flange, with respect to time: (**a**) shear-lag coefficient at the inside, (**b**) shear-lag coefficient at the outside.

The shear-lag coefficient, *λ*, is introduced to reflect the influence of the shear-lag effect of curved composite beams. The shear-lag coefficient, *λ*, is defined as ratio of the stress at the intersection of the web and flange to that at the same location when the shear-lag effect is ignored. Based on the curved composite box girder in Section 3, which is used to verify the accuracy of the proposed model, the changes in the shear-lag coefficient of the concrete slab and steel bottom flange with respect to time are calculated by using the proposed finite beam element model, as shown in Figure 7 and Figure 8, respectively. The results show that the shear-lag coefficient at the inside and the outside of the concrete slab gradually diminishes over time. A small change in the shear-lag coefficient at the inside and outside of the steel bottom flange occurs in the curved composite beams over time, and it tends to stabilize in the late stage of development. The effects of concrete shrinkage and creep can weaken the shear-lag effects in curved composite box girders.

### 4.2. Influence of Time-Dependent Effects on Mechanical Behavior for Different Initial Curvatures

**Figure 9:**
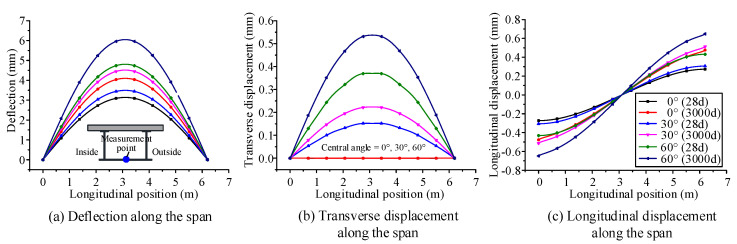
Displacement distribution along the span of the curved composite box girder, with different central angles at the initial loading age and ultimate loading age: (**a**) deflection along the span, (**b**)transverse displacement along the span, (**c**) longitudinal displacement along the span.

**Figure 10:**
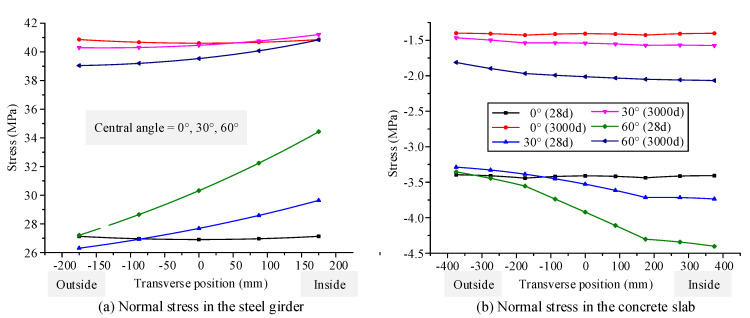
Normal stress distribution along the transverse direction of the curved composite box girder, with different central angles at the initial loading age and ultimate loading age: (**a**) normal stress in the steel girder, (**b**) normal stress in the concrete slab.

The initial central angles are set as 0°, 30°, and 60°, based on the curved composite box girder in Section 3, which is applied to verify the accuracy of the model. According to the proposed finite beam element model, the deflection, transverse displacement, and longitudinal displacement distributions along the span are calculated for a curved composite box girder at the initial and ultimate loading ages, as shown in Figure 9. The normal stress distributions along the transverse direction are calculated for the concrete slab and steel bottom flange at the initial and ultimate loading ages, as shown in Figure 10. Figure 9 shows that the maximum growth rates of deflection for central angles of 0°, 30°, and 60° are 30.5%, 29.2%, and 25.8%, respectively. The maximum growth rates of longitudinal displacement for central angles of 0°, 30°, and 60° are 20.3%, 20.6%, and 21.6%, respectively. The maximum growth rates of transverse displacement of the curved composite box girder with central angles of 30° and 60° are 47.2% and 45.1%, respectively. Figure 10 illustrates the stress distributions of the curved composite box girder with central angles of 0°, 30°, and 60° due to the influence of concrete shrinkage and creep. The tensile stress growth rates at the inside of the steel bottom flange for central angles of 0°, 30°, and 60° are 50.6%, 39.0%, and 18.6%, respectively. The compressive stress reduction rates at the inside of the concrete slab for central angles of 0°, 30°, and 60° are 58.8%, 57.8%, and 53.0%, respectively.

It can be concluded that the growth rate of displacement, caused by concrete shrinkage and creep effect, becomes small with the increase in the initial curvature of the curved box girder. The tensile stress growth rate of the steel girder decreases gradually with increasing initial curvature. The compressive stress reduction rate of the concrete slab decreases gradually with increasing initial curvature.

### 4.3. Influence of the Time-Dependent Effects on Mechanical Behavior for Different Interfacial Shear Connection Stiffness Values

**Figure 11:**
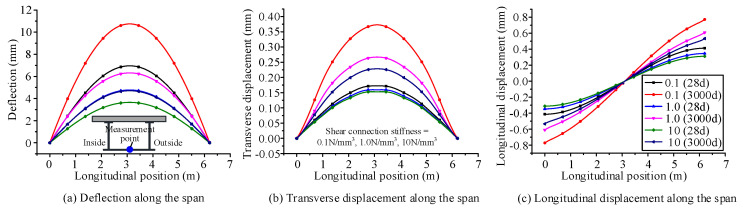
Displacement distribution along the span of curved composite box beam at the initial and ultimate loading ages for different interfacial shear connection stiffness: (**a**) deflection along the span, (**b**) Transverse displacement along the span, (**c**) longitudinal displacement along the span.

**Figure 12:**
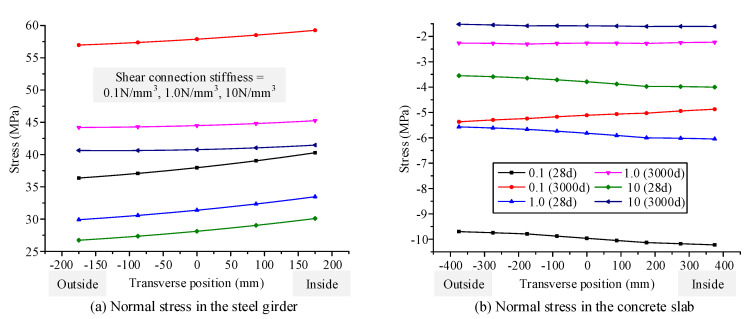
Normal stress distribution along the transverse direction of the curved composite box girder at the initial and ultimate loading ages for different interfacial shear connection stiffness: (**a**) normal stress in the steel girder, (**b**) normal stress in the concrete slab.

The curved composite box girder in Section 3 was selected as the research object. The interfacial shear connection stiffness values are set as 0.1, 1, and 10 N/mm^3^. According to the proposed finite beam element model, the deflection and transverse and longitudinal displacement distributions along the span of the curved composite box girder at the initial and ultimate loading ages are calculated, as shown in Figure 11. The normal stress distributions along the transverse direction are calculated for the concrete slab and steel bottom flange at the initial loading age and ultimate loading age, as shown in Figure 12. Figure 11 shows that the maximum deflection growth rates are 54.4%, 32.3%, and 28.9% for curved composite box girders with shear connection stiffness values of 0.1, 1, and 10 N/mm^3^, respectively, due to the influence of concrete shrinkage and creep. Moreover, for shear connection stiffness values of 0.1, 1, and 10 N/mm^3^, the maximum growth rates of transverse displacement are 116.1%, 67.2%, and 49.2%, respectively, whereas the maximum growth rates of longitudinal displacement are 86.9%, 75.8%, and 71.3%, respectively. Figure 12 shows that the tensile stress growth rates at the inside of the steel bottom flange are 47.1%, 35.2%, and 37.8% for curved composite box girders with shear connection stiffness values of 0.1, 1, and 10 N/mm^3^, respectively, due to the influence of concrete shrinkage and creep. Under these same stiffness values, the compressive stress reduction rates at the inside of the concrete slab are 44.6%, 59.4%, and 57.1%, respectively.

It can be concluded that the growth rate of displacement, which is caused by concrete shrinkage and creep, becomes small as the interfacial shear connection stiffness of the curved composite box girder increases. The tensile stress growth rate of the steel girder decreases with increasing interfacial shear connection stiffness. The compressive stress reduction rate of the concrete slab becomes large with increasing interfacial shear connection stiffness.

## 5. Conclusions

(1) Based on the Vlasov’s one-dimensional theoretical model of curved beams, a torsional warping function, a distortional angle, transverse and longitudinal slip functions, a shear-lag intensity function for the concrete slab, a shear-lag intensity function for the steel beam, and constitutive relationships for concrete shrinkage and creep were introduced. The variation of the curvature in the radial direction was also taken into account. The equilibrium equation for the curved composite box beam was proposed according to the virtual work principle. Then, the stiffness matrix and equivalent nodal load vector of the two-node finite beam element with 26 DOFs, which analyzes the long-term behavior of curved composite box beams, were obtained by adopting the finite element method and the age-adjusted effective modulus method.

(2) An elaborate finite element model of a typical curved composite box beam was established. The correctness and applicability of the two-node finite beam element with 26 DOFs were verified through a comparison of the results from the finite beam element model and those from the elaborate finite element model.

(3) The proposed finite beam element model was adopted to analyze the influence of the concrete time-dependent effects on the long-term behavior of curved composite box beams. The analysis shows that significant changes of the displacement, stress, and shear-lag coefficient occur in the curved composite beams within the first year of loading, due to the time-dependent effects of concrete, after which the variation tendency becomes gradual.

(4) The proposed finite beam element model was adopted to analyze the influence of the concrete shrinkage and creep effects on the long-term behavior of curved composite box beams under different central angles and shear connection stiffness. The results show that increases of the central angle and the shear connection stiffness reduce the change rates of both displacement and stress, with respect to time.

## Appendix A

A quadratic polynomial is used to characterize the shear-lag warping shape function, *ψ*_c_(*x*), of the concrete slab and the shear-lag warping shape function, *ψ*_s_(*x*), of the steel beam, which are expressed in Equations (A1) and (A2), respectively. The physical meaning of the polynomial is the shear-lag warping displacement at any position on the section of the concrete slab or steel beam when a unit shear-lag warping intensity occurs.

(A1) $$\psi_{c}(x)=\left\{ \begin{array}{ll} \left[1-{\left(\frac{x+b_{c}}{b_{c}-b_{t}}\right)}^{2}\right]{\left(\frac{b_{c}-b_{t}}{b_{t}}\right)}^{2} & x>b_{t}\\ 1-{\left(\frac{x}{b_{t}}\right)}^{2} & -b_{t}\leq x\leq b_{t}\\ \left[1-{\left(\frac{x-b_{c}}{b_{c}-b_{t}}\right)}^{2}\right]{\left(\frac{b_{c}-b_{t}}{b_{t}}\right)}^{2} & x<-b_{t} \end{array}\right.$$

(A2) $$\psi_{s}(x)=\left\{ \begin{array}{ll} 0 & \;\mathrm{top}\;\mathrm{flange}\;\mathrm{and}\;\mathrm{web}\;\mathrm{of}\;\mathrm{the}\;\mathrm{steel}\;\mathrm{girder}\\ 1-{\left(\frac{x}{b_{s}}\right)}^{2} & \;\mathrm{bottom}\;\mathrm{flange}\;\mathrm{of}\;\mathrm{the}\;\mathrm{steel}\;\mathrm{girder}\; \end{array}\right.$$

## Appendix B

(1) The nonzero elements of matrix ***B_k_*** are shown hereafter:

*B_k_*(1,1) = −*k*_0_; *B_k_*(1,8) = 1; *B_k_*(1,9) = −*y*_s_*k*_0_; *B_k_*(1,13) = −*y*_d_*k*_0_Ψ_d_; *B_k_*(1,16) = −*a_k_k*_0_/2; *B_k_*(1,19) = *a_k_*/2;

*B_k_*(2,3) = −1; *B_k_*(2,8) = −*k*_0_; *B_k_*(2,19) = −*a_k_k*_0_/2;

*B_k_*(3,6) = −1; *B_k_* (3,9) = *k*_0_; *B_k_*(3,13) = *k*_0_Ψ_d_;

*B_k_*(4,6) = −*k*_0_; *B_k_*(4,12) = −1;

*B_k_*(5,15) = −1;

*B_k_*(6,21) = 1;

*B_k_*(7,23) = 1;

*B_k_*(8,5) = *k*_0_; *B_k_*(8,10) = 1;

*B_k_*(9,5) = −*k*_0_; *B_k_*(9,11) = −1;

*B_k_*(10,20) = 1; and

*B_k_*(11,22) = 1.

In these elements, *k* = s,c represents the steel girder and concrete slab, respectively.

(2) The nonzero elements of matrix ***B*_Ω_** are given hereafter:

*B*_Ω_(1,16) = 1 and *B*_Ω_(2,18) = 1.

## Appendix C

The nonzero elements of the shape function matrix ***N*** are presented hereafter:

*N*(1,1) = *n*_1_; *N*(1,2) = *n*_2_; *N*(1,14) = *n*_3_; *N*(1,15) = *n*_4_;

*N*(2,1) = *n*_1_′; *N*(2,2) = *n*_2_′; *N*(2,14) = *n*_3_′; *N*(2,15) = *n*_4_′;

*N*(3,1) = *n*_1_″; *N*(3,2) = *n*_2_″; *N*(3,14) = *n*_3_″; *N*(3,15) = *n*_4_″;

*N*(4,3) *= n*_1_; *N*(4,4) = *n*_2_; *N*(4,16) = *n*_3_; *N*(4,17) = *n*_4_;

*N*(5,3) = *n*_1_′; *N*(5,4) = *n*_2_′; *N*(5,16) = *n*_3_′; *N*(5,17) = *n*_4_′;

*N*(6,3) = *n*_1_″; *N*(6,4) = *n*_2_″; *N*(6,16) = *n*_3_″; *N*(6,17) = *n*_4_″;

*N*(7,5) = *m*_1_; *N*(7,18) = *m*_2_;

*N*(8,5) = *m*_1_′; *N*(8,18) = *m*_2_′;

*N*(9,6) = *m*_1_; *N*(9,19) = *m*_2_;

*N*(10,6) = *m*_1_′; *N*(10,19) = *m*_2_′;

*N*(11,7) = *m*_1_; *N*(11,20) = *m*_2_;

*N*(12,7) = *m*_1_′; *N*(12,20) = *m*_2_′;

*N*(13,8) = *n*_1_; *N*(13,9) = *n*_2_; *N*(13,21) = *n*_3_; *N*(13,22) = *n*_4_;

*N*(14,8) = *n*_1_′; *N*(14,9) = *n*_2_′; *N*(14,21) = *n*_3_′; *N*(14,22) = *n*_4_′;

*N*(15,8) = *n*_1_″; *N*(15,9) = *n*_2_″; *N*(15,21) = *n*_3_″; *N*(15,22) = *n*_4_″;

*N*(16,10) = *m*_1_; *N*(16,23) = *m*_2_;

*N*(17,10) = *m*_1_′; *N*(17,23) = *m*_2_′;

*N*(18,11) = *m*_1_; *N*(18,24) = *m*_2_;

*N*(19,11) = *m*_1_′; *N*(19,24) = *m*_2_′;

*N*(20,12) = *m*_1_; *N*(20,25) = *m*_2_;

*N*(21,12) = *m*_1_′; *N*(21,25) = *m*_2_′;

*N*(22,13) = *m*_1_; *N*(22,26) = *m*_2_;

*N*(23,13) = *m*_1_′; and *N*(23,26) = *m*_2_′.

The nonzero elements of the shape function matrix ***N*_F_** are given hereafter:

*N*_F_(1,1) = *n*_1_; *N*_F_(1,2) = *n*_2_; *N*_F_(1,14) = *n*_3_; *N*_F_(1,15) = *n*_4_;

*N*_F_(2,3) = *n*_1_; *N*_F_(2,4) = *n*_2_; *N*_F_(2,16) = *n*_3_; *N*_F_(2,17) = *n*_4_;

*N*_F_(3,5) = *m*_1_; *N*_F_(3,18) = *m*_2_;

*N*_F_(4,6) = *m*_1_; *N*_F_(4,19) = *m*_2_;

*N*_F_(5,7) = *m*_1_; *N*_F_(5,20) = *m*_2_;

*N*_F_(6,8) = *n*_1_; *N*_F_(6,9) = *n*_2_; *N*_F_(6,21) = *n*_3_; *N*_F_(6,22) = *n*_4_;

*N*_F_(7,10) = *m*_1_; *N*_F_(7,23) = *m*_2_;

*N*_F_(8,11) = *m*_1_; *N*_F_(8,24) = *m*_2_;

*N*_F_(9,12) = *m*_1_; *N*_F_(9,25) = *m*_2_;

*N*_F_(10,13) = *m*_1_; and *N*_F_(10,26) = *m*_2_.

In these elements, $n_{1}=\left(1+\frac{2z}{l_{e}}\right){\left(\frac{z-l_{e}}{l_{e}}\right)}^{2}$; $n_{2}=z{\left(\frac{z-l_{e}}{l_{e}}\right)}^{2}$; $n_{3}=\left(1-\frac{2\left(z-l_{e}\right)}{l_{e}}\right){\left(\frac{z}{l_{e}}\right)}^{2}$; $n_{4}=\left(z-l_{e}\right){\left(\frac{z}{l_{e}}\right)}^{2}$; $m_{1}=1-\frac{z}{l_{e}}$; and $m_{2}=\frac{z}{l_{e}}$.
